# Glutaminyl cyclase activity correlates with levels of Aβ peptides and mediators of angiogenesis in cerebrospinal fluid of Alzheimer’s disease patients

**DOI:** 10.1186/s13195-017-0266-6

**Published:** 2017-06-06

**Authors:** Claire Bridel, Torsten Hoffmann, Antje Meyer, Sisi Durieux, Marleen A. Koel-Simmelink, Matthias Orth, Philip Scheltens, Inge Lues, Charlotte E. Teunissen

**Affiliations:** 1 Neurochemistry Lab and Biobank, Department of Clinical Chemistry, VU University Medical Centre Amsterdam, Amsterdam, The Netherlands; 2 Probiodrug AG, Halle (Saale), Germany; 3 Swiss BioQuant AG, Basel, Switzerland; 4 Department of Neurology, Alzheimer Center, VU University Medical Centre Amsterdam, Amsterdam, The Netherlands

**Keywords:** Alzheimer’s disease, Cerebrospinal fluid, Glutaminyl cyclase, Amyloid beta, 3pE-Aβ42

## Abstract

**Background:**

Pyroglutamylation of truncated Aβ peptides, which is catalysed by enzyme glutaminyl cyclase (QC), generates pE-Aβ species with enhanced aggregation propensities and resistance to most amino-peptidases and endo-peptidases. pE-Aβ species have been identified as major constituents of Aβ plaques and reduction of pE-Aβ species is associated with improvement of cognitive tasks in animal models of Alzheimer’s disease (AD). Pharmacological inhibition of QC has thus emerged as a promising therapeutic approach for AD. Here, we question whether cerebrospinal fluid (CSF) QC enzymatic activity differs between AD patients and controls and whether inflammatory or angiogenesis mediators, some of which are potential QC substrates, and/or Aβ peptides may serve as pharmacodynamic read-outs for QC inhibition.

**Methods:**

QC activity, Aβ peptides and inflammatory or angiogenesis mediators were measured in CSF of a clinically well-characterized cohort of 20 mild AD patients, 20 moderate AD patients and 20 subjective memory complaints (SMC) controls. Correlation of these parameters with core diagnostic CSF AD biomarkers (Aβ42, tau and p-tau) and clinical features was evaluated.

**Results:**

QC activity shows a tendency to decrease with AD progression (*p* = 0.129). The addition of QC activity to biomarkers tau and p-tau significantly increases diagnostic power (ROC-AUC_TAU_ = 0.878, ROC-AUC_TAU_&_QC_ = 0.939 and ROC-AUC_pTAU_ = 0.820, ROC-AUC_pTAU_&_QC_ = 0.948). In AD and controls, QC activity correlates with Aβ38 (*r* = 0.83, *p* < 0.0001) and Aβ40 (*r* = 0.84, *p* < 0.0001), angiogenesis mediators (Flt1, Tie2, VEGFD, VCAM-1 and ICAM-1, *r* > 0.5, *p* < 0.0001) and core diagnostic biomarkers (*r* > 0.35, *p* = <0.0057). QC activity does not correlate with MMSE or ApoE genotype.

**Conclusions:**

Aβ38, Aβ40 and angiogenesis mediators (Flt1, Tie2, VEGFD, VCAM-1 and ICAM-1) are potential pharmacodynamic markers of QC inhibition, because their levels closely correlate with QC activity in AD patients. The addition of QC activity to core diagnostic CSF biomarkers may be of specific interest in clinical cases with discordant imaging and biochemical biomarker results.

**Electronic supplementary material:**

The online version of this article (doi:10.1186/s13195-017-0266-6) contains supplementary material, which is available to authorized users.

## Background

Soluble, non-fibrillar amyloid-beta (Aβ) oligomers are key players in the initiation and propagation of Alzheimer’s disease (AD) pathology [1]. Variable amyloid precursor protein (APP) cleavage by β-secretases and γ-secretases generates a diversity of Aβ species spanning 34–50 amino acids in length [2, 3]. Further variability arises from N-terminal truncations of these peptides [4, 5], and from N-terminal post-translational modifications including pyroglutamylation, isomerization and racemization [2]. Pyroglutamylation, a dehydration reaction converting N-terminal glutamate (E) into pyroglutamate (pE), generates pE-Aβ species with enhanced aggregation propensities [6–8] and resistance to most amino-peptidases and endo-peptidases [9]. pE-Aβ species have been identified as major constituents of Aβ plaques [10–12]. One of these, 3pE-Aβ42, has gained much attention over the past years since the demonstration of its ability to trigger neurodegeneration when expressed transgenically in mouse [13] and to seed aggregation of Aβ species to form highly toxic Aβ oligomers both in vitro and in vivo [14].

Pyroglutamylation of truncated Aβ species carrying a glutamate residue at position 3 or 11 (including Aβ3-40, Aβ11-40, Aβ3-42 and Aβ11-42) is catalysed by enzyme glutaminyl cyclase (QC) [15–17], whose expression is increased in temporal and enthorinal cortices of AD patients and correlates with insoluble pE-Aβ aggregates [18]. In addition to N-terminal glutamate Aβ species, QC catalyses pyroglutamation of a number of other substrates harbouring an N-terminal glutamine residue, including inflammatory mediators monocyte chemoattractant proteins (MCP) 1–4 (also known as CCL2, CCL8, CCL7 and CCL13) [19] and possibly fractalkine (CX3CL1) (Kehlen et al., manuscript in preparation), C-reactive protein (CRP) [20] and soluble ICAM-1 [19, 21]. These may be of particular relevance to AD pathomechanisms, because local chronic inflammation resulting from microglia and astrocyte activation by Aβ aggregates is a well-described feature of AD pathology [22–24] and affects APP and Aβ processing [25–27].

In mouse, chronic pharmacological inhibition or genetic ablation of QC results in reduced pE-Aβ brain levels and improvement of cognitive tasks in models of AD [16]. Reduction of pE-Aβ levels through pharmacological inhibition of QC has thus emerged as a promising therapeutic approach for AD. QC inhibitor PQ912 was reported safe and well tolerated in a recent phase 1 study [28]. In young (18–50 years old) healthy individuals, PQ912 reduced both serum and cerebrospinal fluid (CSF) QC activity in a dose-dependent manner [28]. In the present study, we first measured QC enzymatic activity in CSF of AD patients and questioned whether it differed from that of age-matched controls, offering a prospect as a diagnostic or disease progression biomarker. Second, we set out to measure CSF levels of Aβ peptides, as well as angiogenesis and inflammatory mediators, whose levels may be modified in AD CSF, given the involvement of angiogenesis and inflammation in AD pathomechanisms [24, 29]. All parameters were analysed for differences between diagnosis groups and correlation with QC activity.

## Methods

### Patients

All patients and corresponding CSF samples were selected from the Alzheimer Center Memory Cohort, NeuroUnit Biomarkers for Inflammation and Neurodegeneration, VU Medical Center (VUmc) Biobank (Amsterdam, the Netherlands). We included 40 patients with probable AD and 20 patients with subjective memory complaints (SMC), which served as a control group. All patients underwent extensive dementia screening at baseline, including physical and neurological examination, EEG, MRI and laboratory tests. Neuropsychological assessment was performed and included the Mini Mental State Examination (MMSE) for global cognition. Diagnoses were made by consensus in a multidisciplinary meeting. Probable AD was diagnosed according to the core clinical National Institute on Aging–Alzheimer's Association (NIA-AA) criteria [30]. Mild AD (*n* = 20) is defined here as patients with MMSE score > 18 and < 24, and moderate AD (*n* = 20) as patients with MMSE score ≤ 18 and ≥ 15. The cohort selection was based on short CSF storage time to avoid storage artefacts (mean storage time ± SEM: 2.92 ± 1.45 years) and MMSE score. Core CSF diagnostic biomarkers (Aβ42, tau and p-tau) were not part of the diagnostic workup. Diagnosis of SMC was determined when results of all clinical examinations were normal, and there was no psychiatric diagnosis. The study was approved by the local ethical review board and all subjects gave written informed consent for the use of their clinical data for research purposes.

### CSF collection

CSF was obtained by lumbar puncture using a 25-gauge needle, and collected in 10-ml polypropylene tubes (Sarstedt, Nümbrecht, Germany). Within 2 hours of collection, CSF was centrifuged at 1800 × *g* for 10 min at 4 °C, transferred to new polypropylene tubes and stored at −80 °C until further analysis [31]. CSF was analysed without knowledge of clinical diagnoses.

### Core CSF diagnostic AD biomarkers

Core diagnostic AD biomarkers tau and p-tau were measured in CSF by commercially available ELISAs (Innotest hTAU-Ag and Innotest Phosphotau (181P); Innogenetics, Ghent, Belgium), according to the manufacturer’s instructions at the Neurochemistry Laboratory, VUmc. Core diagnostic AD biomarker Aβx-42 as well as C-terminally truncated Aβ variants Aβx-38 and Aβx-40 were measured using two commercially available multiplex immunoassays (Abeta 3-Plex Kit 6E10 and 4G8; Meso Scale Diagnostics, Rockville, USA). The first multiplex assay makes use of monoclonal antibody 6E10 (Covance; reactive to Aβ1-16, binding epitope between amino acids 4 and 9) as the capture antibody, and peptide-specific detection antibodies. The second multiplex assay makes use of monoclonal antibody 4G8 (Covance; reactive to amino acids 17–24 of Aβ) as the capture antibody, and peptide-specific detection antibodies. Method correlation between the 4G8 multiplex immunoassay and the 6E10 multiplex immunoassay was strong (see Additional file 1: Figure S2).

### QC activity in CSF

QC activity in CSF was measured as described previously [28, 32] by a flourimetric assay using Gln-AMC as the substrate and pyroglutamyl aminopeptidase as the auxiliary enzyme, and was performed by Evotec AG (Hamburg, Germany).

### Truncated Aβ peptides in CSF

N-terminal variants of Aβ peptides Aβ3-40, Aβ11-40 and Aβ11-42 were quantified by liquid chromatography tandem mass spectrometry (LC-MS/MS). A suitable LC-MS/MS approach for absolute quantification of Aβ1-40 and Aβ1-42 was described previously [33] and is currently under validation as a standard reference method [34, 35]. Method correlation between the multiplex immunoassays and  LC-MS/MS for Aβ quantification is shown in Additional file 2: Figure S3. We applied and further adapted this analytical approach by including also the N-terminally truncated peptides Aβ3-40, Aβ11-40, Aβ3-42 and Aβ11-42. In contrast to Lame et al. [33], and due to the much lower abundance of the N-terminally truncated peptides, we used immuno-affinity enrichment instead of solid-phase extraction to extract the peptides from CSF.

A previously described method by Kleinschmidt et al. [36] for Aβ peptide extraction from human plasma using a mixture of three anti-Aβ monoclonal antibodies was modified for the extraction of 100 μl CSF. Equal aliquots of 4G8, 12 F4 and 11A50-B10 antibodies (2 μg; supplied by Hiss Diagnostics, Germany) and stable isotope-labelled internal standards (1 ng/ml final concentration, Additional file 3: Table S1) were added to 100 μl CSF in 400 μl phosphate-buffered saline (PBS) containing 1% BSA and 0.05% Tween. After an overnight incubation on a rotary shaker at 4 °C, the immuno-complex was coupled to pre-washed Dynabeads™ M-280 Sheep Anti-Mouse IgG (Invitrogen) for another 2 hours at 25 °C. The beads were separated magnetically, washed with PBS and twice with 25 mM ammonium bicarbonate containing 0.1% octyl β-d-glucopyranoside and the peptides of interest were eluted using 100 μl of 50% acetonitrile containing 1% ammonium hydroxide (50/50, v/v). Then 20-μl extracts were injected into an XBridge BEH130 C18 column (3.5 μm, 2.1 mm × 150 mm; Waters) operating at 50 °C using an UltiMate 3000 RSLC nano system (Dionex) (for a chromatogram of a sample extract, see Additional file 4: Figure S1). The mobile phase consisted of 0.1% ammonium hydroxide/acteonitrile (95/5, v/v) (solvent A) and 0.1% ammonium hydroxide (5/95, v/v) (solvent B). A linear gradient at a flow rate of 400 μl/min starting from 2% solvent B to 70% solvent B was applied for 3.5 min with a further increase to 100% B before re-equilibration. The LC was coupled to a TSQ Quantiva Triple Quadrupole mass spectrometer (Thermo Fisher Scientific) operating in selected reaction monitoring (SRM) positive mode using heated electrospray ionization.

Because absolute quantification depends on the quality of the reference items, we and others [35] discovered that determining the exact peptide content of Aβ peptides appears to be difficult, due to potential chemical modifications, non-specific binding and a tendency for aggregation. Therefore, a relative quantification approach, considering the relative response of analyte versus internal standard, was used within this study. Nevertheless, calibration curves from spike-in of reference peptides into artificial CSF were prepared for each analytical batch and found to be linear within the anticipated concentration range. Reference peptides were synthesized by JPT Peptide Technologies GmbH (Germany) and Innovagen (Sweden). To assess the inter-assay variability, an endogenous CSF pool was prepared and spiked with internal standard peptides, stored at −80 °C and analysed as a quality control sample within each analytical batch. The respective response ratios were stable over a month’s storage (Additional file 2: Table S2).

### Inflammatory mediators

CSF levels of inflammatory mediators were assessed using commercially available 37-plex ELISAs (Mesoscale Discovery, MSD) according to the manufacturer’s instructions, which measure panels of proinflammatory molecules (IFN-γ, IL-1β, IL-2, IL-4, IL-6, IL-8, IL-10, IL-13, TNFα), chemokines (Eotaxin, Eotaxin-3, IP10, MCP-1, MCP-4, MDC, MIP-1α, MIP-1β, TARC), cytokines (IL1-α, IL-5, IL-7, IL-12, IL-15, IL-16, IL-17A, TNF-β, VEGF), angiogenesis mediators (bFGF, Flt1, PlGF, Tie2, VEGF-D, VEGF-C) and vascular injury markers (SAA, CRP, VCAM-1, ICAM-1).

### Statistical analysis

R version 3.0.1, including packages “pgirmes”, “pROC” and “ellipse”, were used for statistical analysis and figure preparation. All biochemical CSF markers which were not normally distributed (Shapiro–Wilk test) were log-transformed before correlation analyses to achieve normal distribution. Pearson’s correlation coefficient (*r*) and the respective *p* values are provided in the figures. Groups were compared for all variables using the Kruskal–Wallis test. For each parameter, correction for multiple comparisons between groups was done using the function “kruskalmc” (“pgirmess” package) after the Kruskal–Wallis test. *p* <0.05 was considered significant. Because of the exploratory character of our study, no further correction for multiple testing was performed.

## Results

Baseline characteristics and apolipoprotein E (ApoE) genotype distribution of the study population are presented in Table 1. Age was comparable between groups. E4/E4 and E3/E4 genotypes were slightly more frequent in mild AD, but Fisher’s exact test revealed no significant differences in ApoE genotype distribution between groups (*p* = 0.55).

**Table 1:** Demographics and ApoE genotype proportion of cohort

Diagnosis	*N*	% female	Mean (SD) age (years)	Mean (SD) MMSE score	ApoE genotype, *N* (%)		
					E3E3	E3E4	E4E4
Mild AD	20	35.7	60.4 (2.9)	21.1 (1.0)	9 (45%)	10 (50%)	1 (5%)
Moderate AD	20	61.5	60.1 (2.8)	15.1 (2.0)	5 (25%)	12 (60%)	3 (15%)
SMC	20	42.0	60.3 (2.7)	26.9 (1.9)	9 (47%)	8 (42%)	2 (11%)

*AD* Alzheimer’s disease, *SMC* subjective memory complaints, *MMSE* Mini Mental Status Evaluation, *ApoE* apolipoprotein E

### Levels of core diagnostic AD biomarkers, C-terminally truncated Aβ variants and QC activity in CSF of AD patients

**Fig. 1:**
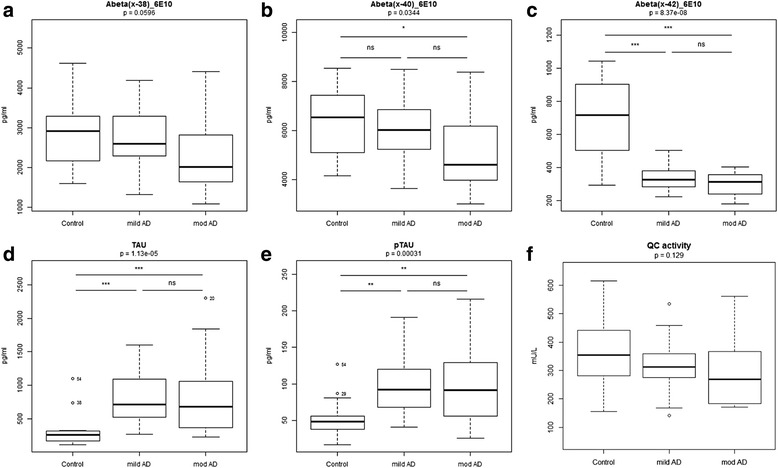
Levels of Alzheimer’s disease (*AD*) core CSF biomarkers and QC activity in CSF of mild and moderate AD patients compared with control subjects (SMC). Core CSF biomarkers **a** Aβ38, **b** Aβ40, **c** Aβ42, **d** Tau and **e** ptau. **f** QC activity. *p* values from Kruskal–Wallis statistics, *n* = 20 per group, post-hoc test results: *ns* not significant, *p < 0.05, **p < 0.01, ***p < 0.001, outliers marked with patient number

**Fig. 2:**
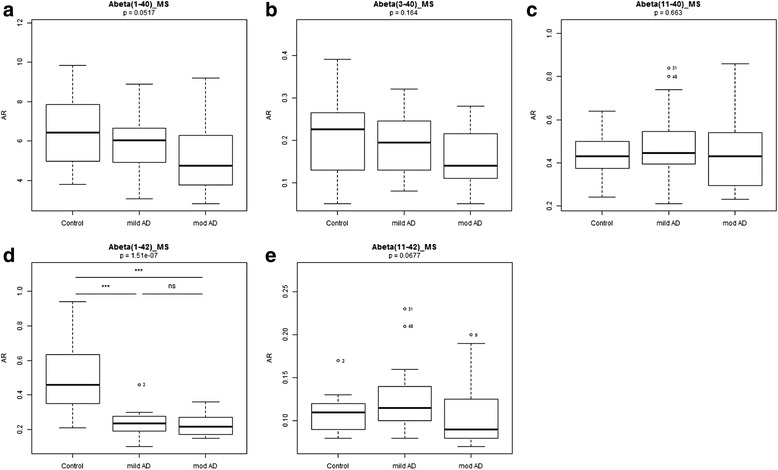
CSF levels of N-terminal variants of Aβ peptides determined by LC-MS. **a** Aβ1-40, **b** Aβ3-40, **c** Aβ11-40, **d** Aβ1-42 and **e** Aβ11-42. *p* values from Kruskal–Wallis statistics, post-hoc test results: *ns* not significant, ***p < 0.001, outliers (> median ± 1.5 × IQR) marked with patient number. *AR* analyte versus internal standard peak area ratio, *Aβ* amyloid beta, *AD* Alzheimer’s disease

Core diagnostic AD CSF biomarker (Aβ42, tau and p-tau) levels were distributed as expected across the three diagnosis groups: significantly lower levels of Aβ42 in AD patients and significantly higher levels of tau and p-tau in AD patients compared with SMC controls (Fig. 1c–e). C-terminally truncated Aβ variants Aβ38 and Aβ40 showed similar trends towards decrease with progression of AD, reaching significance between moderate AD and controls for Aβ40 only (*p* < 0.05, Fig. 1a, b). QC activity showed nominally decreased mean activities with AD progression, but without reaching significance (*p* = 0.129, Fig. 1f). In CSF of AD and controls, LC-MS/MS allowed relative quantification of three N-terminal-variants Aβ3-40, Aβ11-40 and Aβ11-42, in addition to full-length peptides Aβ1-40 and Aβ1-42. These truncated peptides all carry an N-terminal glutamate and are proven QC substrates [15, 17]. Aβ3-40 showed a slight decrease of mean levels with progression of AD, without reaching significance (*p* = 0.164) (Fig. 2a, b). A similar trend was observed for Aβ1-40 (*p* = 0.052). Aβ11-40 (*p* = 0.663) and Aβ11-42 (*p* = 0.068) levels showed no differences between diagnosis groups. Aβ11-42 thus clearly differed from Aβ42, whose levels differentiate controls and AD patients (*p* < 0.0001, Fig. 2c–e). Levels of Aβ3-42 and pE modified species could not be detected with the LC-MS/MS method used in this study. Levels of full-length Aβ1-40 and Aβ1-42 determined by LC-MS/MS correlated well with respective Aβ40 and Aβ42 levels determined by ELISA (*r*_Aβ40_ = 0.944 and *r*_Aβ42_ = 0.933, see Additional file 1: Figure S2).

### Correlation of QC activity with core AD biomarkers

**Fig. 3:**
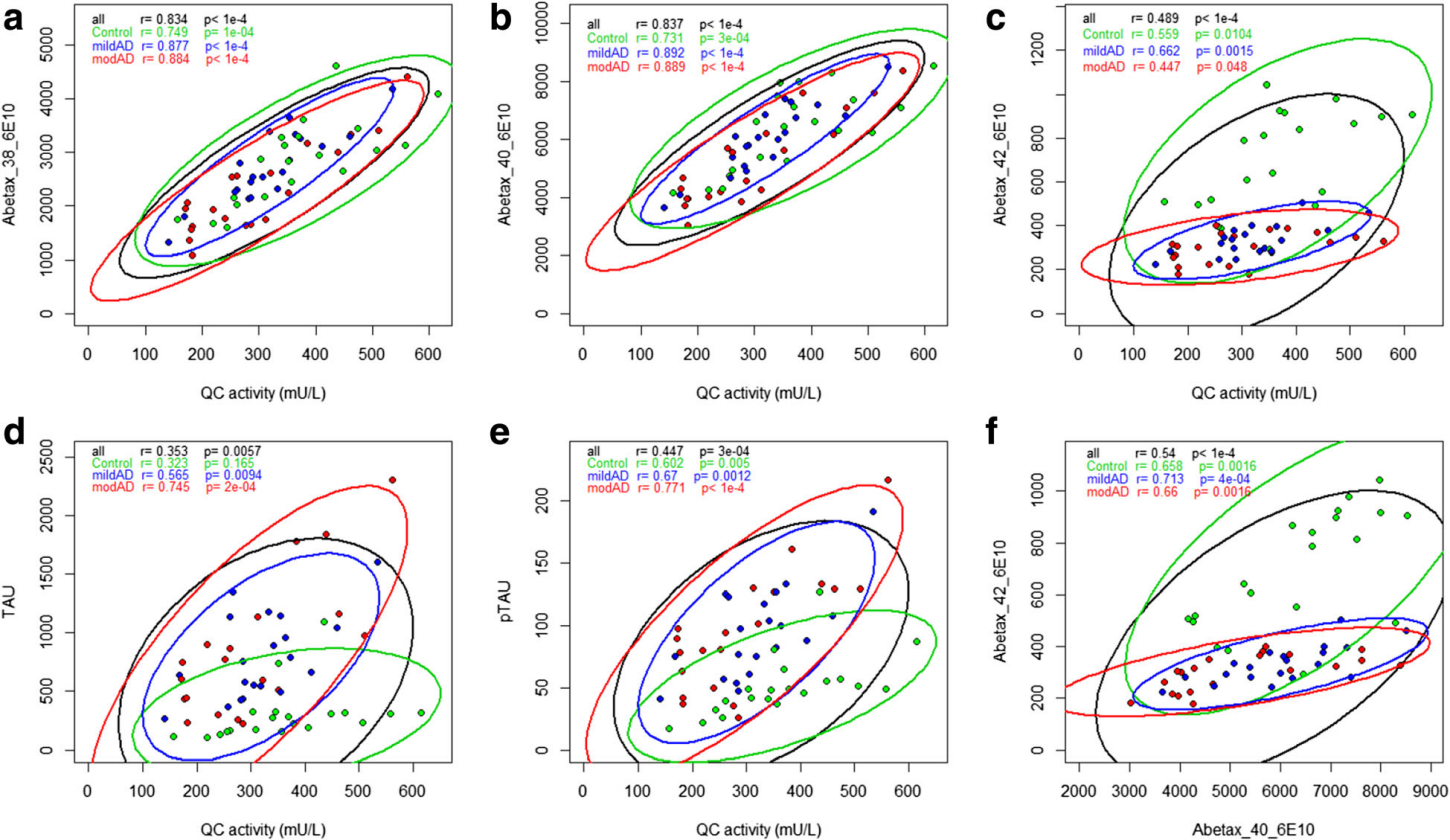
Correlation of CSF QC activity (mU/L) with levels of Aβ (pg/ml) and Tau peptides (pg/ml) and Aβ40–Aβ42 correlation. **a** Aβ38, **b** Aβ40, **c** Aβ42, **d** Tau, **e** ptau and **f** Aβ40–Aβ42 correlation. Different diagnosis groups are colour coded (*green*: control, *blue*: mild AD, *red*: moderate AD). Pearson’s correlation coefficient with *p* values given for each parameter and group. *Ellipses* give 95% CI of the scatter for each group. Width and sloop of ellipses give a visual impression of strength of correlation: *slim ellipses* with sloops clearly different from 0 or 1 characterize high correlation of both parameters whereas *wide ellipses* with sloops narrow to 0 or 1 indicate less or no correlation. *AD* Alzheimer’s disease, *QC* glutaminyl cyclase (Colour figure online)

**Fig. 4:**
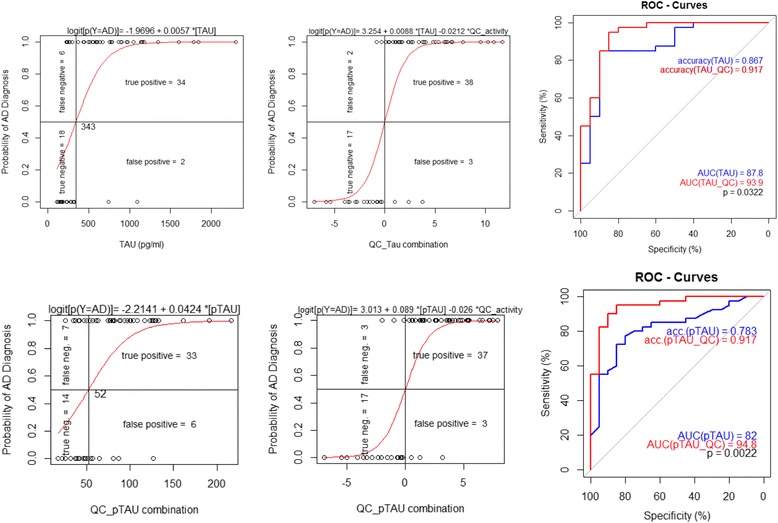
Improvement of diagnostic performance by addition of QC activity to logistic regression for AD prediction. *Upper left*: tau levels (*open circles*) of control (0) and AD (1) groups and logistic regression curve (*red*) with tau as a single parameter. *Upper middle*: logistic regression curve (*red*) calculated with tau and QC as variables and levels of combined tau–QC parameter (*open circles*) for control (0) and AD (1) groups. *Upper right*: ROC curves for results of logistic regression with tau alone (*blue*) or a tau–QC combination (*red*), *p* value from DeLongs test. *Lower left*: ptau levels (*open circles*) of control (0) and AD (1) groups and logistic regression curve (*red*) with ptau as a single parameter. *Lower middle*: logistic regression curve (*red*) calculated with ptau and QC as variables and levels of combined ptau–QC parameter (*open circles*) for control (0) and AD (1) groups. *Lower right*: ROC curves for results of logistic regression with ptau alone (*blue*) or a ptau–QC combination (*red*), *p* value from DeLongs test. *AD* Alzheimer’s disease, *QC* glutaminyl cyclase, *ROC* receiver operating characteristic (Colour figure online)

A high correlation of QC activity was observed with Aβ38 and Aβ40, with Pearson correlation coefficients of 0.83 (*p* < 0.0001) and 0.84 (*p* < 0.0001) respectively (Fig. 3a, b). When considering all study subjects together, the correlations of QC activity with Aβ42, tau and p-tau were moderate, with correlation coefficients below 0.5 (Fig. 3c–e, black lines). However, when evaluating each diagnosis group separately, we observed higher correlations of QC activity with Aβ42 in the control group (*r* = 0.56) and mild AD group (*r* = 0.66) and with tau and p-tau for both AD groups (*r* = up to 0.77 for QC-ptau correlation in moderate AD) (Fig. 3c–e, coloured lines). Most strikingly, for tau and p-tau (Fig. 3d, e) higher correlations as well as a larger increase of tau respective to p-tau per increased unit of QC activity were observed in the AD groups (Fig. 3d, e, blue and red lines) compared with the control group (green lines). This was reflected in a better diagnostic performance of a combination of tau and QC or p-tau and QC compared with tau or p-tau alone. Figure 4 shows logistic regression and receiver operating characteristic (ROC) curves for tau and p-tau and their combination with QC activity. With addition of the QC parameter, the area under the curve (AUC) increases from 87.8 to 93.9% (*p* = 0.032) and from 82.0 to 94.8% (*p* = 0.002) for tau and p-tau respectively.

### Correlation of QC activity with inflammatory parameters

**Fig. 5:**
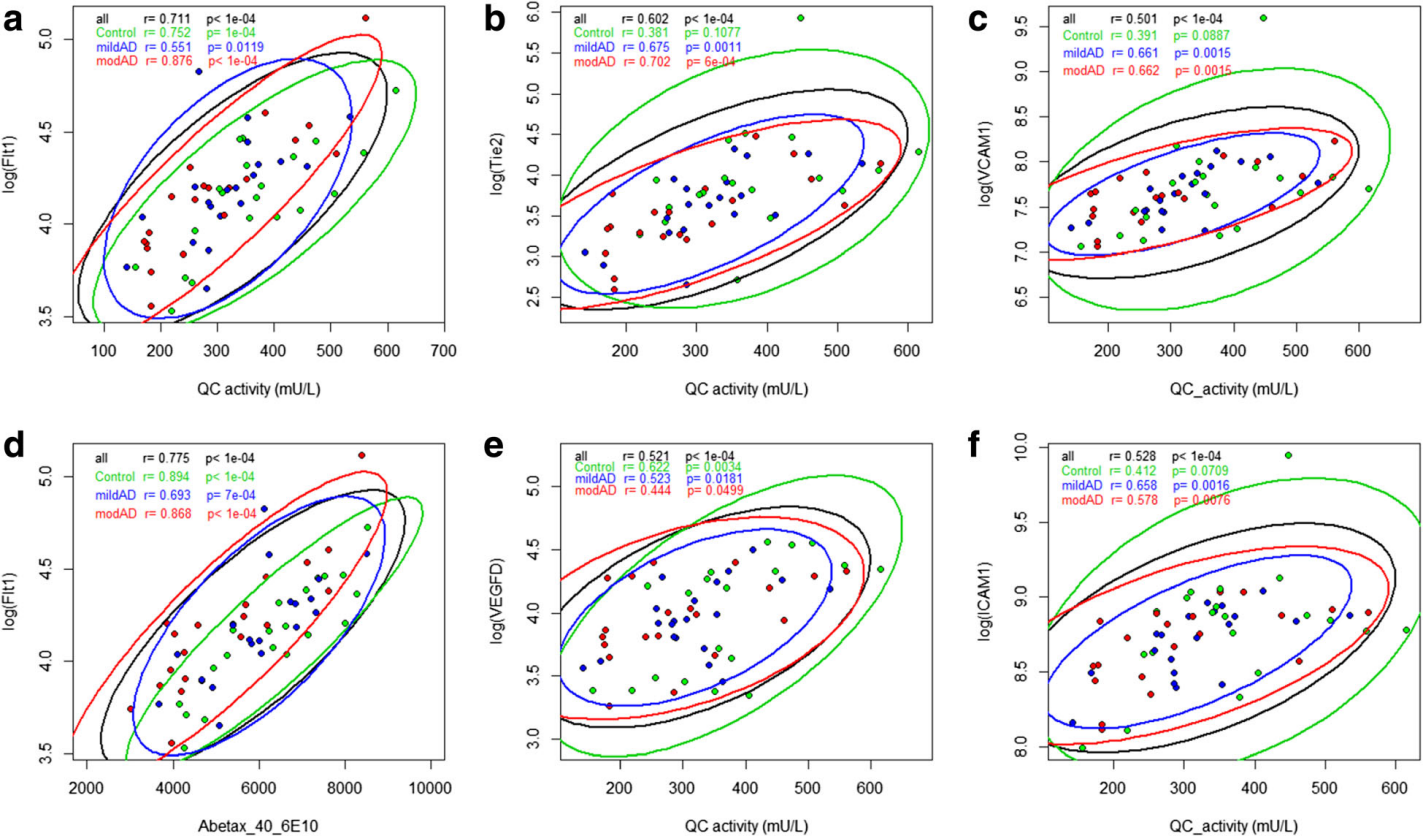
Correlation of CSF QC activity (mU/L) or Aβ40 with log-transformed levels of Flt1, Tie2, ICAM-1, VEGFD and VCAM-1. QC activity with **a** Flt1, **b** Tie2, **c** VCAM-1, **e** VEGFD and **f** ICAM-1. **d** Aβ40 with Flt1. Different diagnosis groups are colour coded (*green*: control, *blue*: mild AD, *red*: moderate AD). Pearson’s correlation coefficient with *p* values given for each parameter and group. *Ellipses* give 95% CI of the scatter for each group. Width and sloop of ellipses give a visual impression of strength of correlation: *slim ellipses* with sloops clearly different from 0 or 1 characterize high correlation of both parameters whereas *wide ellipses* with sloops narrow to 0 or 1 indicate less or no correlation. *AD* Alzheimer’s disease, *QC* glutaminyl cyclase (Colour figure online)

A positive correlation was observed between QC activity and a number of analytes from the angiogenesis panel. In particular, QC activity correlated with angiogenesis mediators Flt1 (*r* = 0.711, *p* < 0.0001), Tie2 (*r* = 0.602, *p* < 0.0001) and VEGF-D (*r* = 0.521, *p* < 0.0001), as well as with soluble adhesion molecules ICAM-1 (*r* = 0.528) and VCAM-1 (*r* = 0.501) (Fig. 5). No differences between the three groups were identified for the remaining inflammatory mediators (IL1-β, IL-6, IL-8, Eotaxin, Eotaxin-3, IP10, MDC, MIP-1α, MIP-1β, TARC, SAA, PIGF) with the exception of IL-5, where a slight decrease was observed in the moderate AD group compared with control (*p* = 0.034). All other investigated inflammatory mediators (VEGF-C, IL-1α, IL-17a, TNF-β, IFN-γ, IL-10, IL-13, IL-2, IL-4, TNF-α) were below the lower limit of detection in more than 1/3 of samples across all three diagnostic groups and were thus not further evaluated (data not shown). QC activity did not correlate with MMSE scores or E4/E4 genotype (data not shown).

## Discussion

In this study including 40 patients with probable AD and 20 controls with SMC, we find that QC activity shows a tendency towards decreased activity with progression of AD and that its addition to biomarkers tau and p-tau significantly increases the diagnostic power of these biomarkers. In AD individuals and controls, QC activity strongly correlates with levels of Aβ38 and Aβ40, as well as with levels of soluble ICAM-1, VCAM-1 and three angiogenesis mediators.

A mean QC activity of 366 ± 116 mU/L (mean ± SD) was measured in CSF of SMC individuals, similar to the mean value of 376 mU/L reported by Lues et al. [28] using the same method in young (18–55 years old) healthy individuals. Although there was no significant difference between SMC and AD, we observed nominally decreased mean QC activities in mild (319 ± 89 mU/L) and moderate (297 ± 120 mU/L) AD compared with age-matched SMC. This tendency towards decreased QC activity in AD contrasts with the increased QC protein expression and pE-Aβ levels reported in temporal and enthorinal cortex of AD patients [18], as well as with the increased QC mRNA levels reported in the brain [37] and blood [38] of AD patients. QC activity has not so far been measured in brain tissue, and the relation between expression level and activity of QC remains to be established. Defining the behaviour of QC activity with AD progression will require a more powered study as well as additional categories of disease severity ranging from MCI to severe AD.

Conflicting results concerning Aβ38 and Aβ40 levels in CSF or plasma of AD patients are reported in the literature, ranging from decreased [39] or increased [40] levels in AD compared with controls to no changes between AD and controls [36, 41]. In our cohort, we observed a trend towards decreased Aβ38 and Aβ40 levels with AD progression, paralleling the tendency for decreased QC activity. In addition, QC activity showed very high positive correlation with Aβ38 and Aβ40, which together reflect the main portion of total Aβ in the brain. This correlation is independent of diagnosis groups, which may point towards a higher level of interdependence or co-regulation of Aβ38, Aβ40 and QC activity. In the literature, the addition of Aβ38 and Aβ40 to core diagnostic biomarkers has been reported to improve discriminative power [36, 39–45]. In our cohort, we found that the addition of QC activity to diagnostic biomarkers tau and p-tau increased diagnostic power compared with the respective biomarkers alone. This finding may result from the high correlation and interdependence of QC activity with Aβ38 and Aβ40, further supporting the idea of a co-regulation of these parameters. It will be interesting to investigate, in clinical trials with AD patients, whether QC inhibition leads to reduced Aβ38 and Aβ40 levels, thus offering potential as pharmacodynamic markers of QC activity and surrogate markers of treatment response. Because Aβ38 and Aβ40 are included in multiplex Aβ panels and can be easily assessed as part of routine AD workup in clinical trials, their assessment may offer practical advantages over QC activity measurement.

Using LC-MS/MS, QC substrates Aβ3-40, Aβ11-40 and Aβ11-42 could be quantified along with full-length peptides Aβ1-40 and Aβ1-42. As for full-length Aβ40, a trend towards decreased levels with AD progression was observed for Aβ3-40, but not for Aβ11-40. In contrast to its full-length counterpart Aβ42, Aβ11-42 levels were similar across all three diagnostic groups, and Aβ11-42 and Aβ42 levels did not correlate. This could indicate differences in solubility or proteolytic pathways involved in the generation of these peptides. Using ELISA on a similar sized cohort, Abraham et al. [46] found lower levels of Aβ11-40 and Aβ11-42 peptides in CSF of mild cognitive impairment (MCI) subjects compared with healthy controls. However, the levels of these peptides did not differ between MCI and AD subjects and AD and controls. Differences in group definition may explain the discrepancies with our findings.

In contrast to the high correlation with Aβ38 and Aβ40, only a moderate positive correlation between QC activity and core diagnostic biomarkers Aβ42, tau or ptau was observed. Correlations were greater when the three diagnosis groups were considered individually rather than pooled, further supporting a biological relevance of QC activity in AD.

QC activity correlated with soluble adhesion molecules ICAM-1 and VCAM-1 and angiogenesis mediators including endothelial cell-specific receptors Tie-2 and Flt1 (a.k.a. VEGFR-1) as well as vascular endothelial growth factor VEGF-D. The latter correlation is of particular relevance, given the increasing evidence linking the VEGF pathway to AD pathology, and angiogenesis being hypothesized to play an active part in AD pathology [29]. A VEGF gene polymorphism has been associated with increased risk of AD [47, 48], and Aβ was reported to block VEGFR-2-mediated signalling [49]. In mouse models of AD, VEGF was shown to increase neurogenesis and restore memory impairment [50–52]. From a biomarker perspective, decreased serum [53–55] and increased CSF [56] VEGF levels have been reported in AD. The correlation between QC activity and VEGF-D raises the possibility that QC inhibition influences this system. Further research is needed to determine whether this holds true and what the consequences may be.

## Conclusions

We find that QC activity shows a tendency towards decrease with AD severity and correlates highly with some Aβ peptides and mediators of angiogenesis. Aβ38, Aβ40, soluble ICAM-1 and VCAM-1, VEGF-D, Tie2 and Flt1 may serve as pharmacodynamic read-outs for QC inhibition and surrogate markers of treatment response, because their levels closely correlate with QC activity in AD patients. Moreover, the addition of these parameters to core diagnostic CSF biomarkers may improve diagnostic power, and be of specific interest in clinical cases with discordant imaging and core biochemical biomarker results.

## Additional files

Additional file 1: Figure S2. showing method comparison for Aβs x-38, x-40 and x-42 determination in CSF of AD patients and SMC: MSD Aβ-6E10 versus MSD Aβ-4G8 multiplex. *Upper panels*: scatter plots with regression line (Deming regression, *blue solid*) and line of identity (*black dashed*). Regression equation with 95% confidence intervals of slope and intercept and coefficient of determination (*R* ^2^) given at top of each figure. *Lower panels*: Bland–Altman plots with line of identity (*solid, black*), bias (*solid, blue*), limits of acceptance (*dashed, blue*) and ±25% deviation between assays (*dotted, black*). Aβ38: regression line slope (1.10 (0.99, 1.22)) is slightly higher but not significantly different from 1, whereas intercept (−561 (−878, −244) pg/ml) is significantly below 0. Aβ 40: slope (1.02 (0.89, 1.14)) and intercept (−466 (−1236, 304) pg/ml) are not significantly different from 1 or 0, respectively. Regression line is parallel but slightly below the line of identity, resulting in slightly lower concentrations determined by the 4G8 assay (about 94% of 6E10 in mean). In the Bland–Altman plot, there is no trend over the concentration range analysed and only four samples show more than 25% lower values compared with the 6E10 assay. Aβ42: regression line slope of 0.92 (0.84, 0.99) is lower than 1 and the intercept of 55 (19, 92) pg/ml is significantly above 0. Therefore, in the low concentration range (<400 pg/ml), higher values of Aβ42 are determined with the 4G8 assay compared with the 6E10 assay. Because of the group difference, this affects most of the AD samples whereas for the control samples very similar values were determined with both assays (see Bland–Altman plot). (TIF 486 kb)

Additional file 2: Figure S3. showing method comparison for Aβ determination in CSF of AD patients and SMC: LC-MS (relative quantification) versus MSD Aβ multiplex. *Upper panels*: scatter plot with Deming regression lines. *Lower panels*, Bland–Altman plots for Aβ40 assay comparison. For Aβ1-42, only relative quantification was done with LC-MS. LC-MS determinations of Aβ(1–40) and Aβ(1–42) show correlation with both Aβ multiplexes. Coefficients of determination were found to be slightly better with the 6E10 multiplex assays. For the Aβ40 assays, Deming regression lines show slope < 1 and cross the lines of identity in the medium concentration range. Compared with the ELISAs, the LC-MS assay underestimates Aβ40 in the low concentration range and overestimates it at high concentrations. Nevertheless, mean deviation is <5% and there were only 5 out of 60 and 2 out of 60 measurements out of the ±25% range compared with the 4G8 multiplex or 6E10 multiplex, respectively. (TIF 484 kb)

Additional file 3: Table S1. presenting a summary of the respective reference Aβ peptides (sequence, molecular weight, charge state, selected SRM transitions and provider. V* = Val (U13C5; 15 N)), and **Table S2** presenting relative response of endogenous Aβx-40 peptides (analyte versus internal standard peak area ratios) extracted from a native CSF sample pool analysed over a storage period of 1 month at −80 °C (*n* = 3). (DOCX 40 kb)

Additional file 4: Figure S1. showing LC-MS/MS chromatogram of endogenous full-length peptides Aβ1-40 and Aβ1-42 and N-terminally truncated peptides Aβ3-40, Aβ11-40 and Aβ11-42 extracted from 100 μl CSF. Aβ3-42 was not detectable (see *lane 5*). (DOCX 85 kb)

## Abbreviations

AD: Alzheimer’s disease
ApoE: Apolipoprotein E
APP: Amyloid precursor protein
Aβ: Amyloid beta
CSF: Cerebrospinal fluid
EEG: Electro-encephalography
MCI: Mild cognitive impairment
MMSE: Mini Mental State Evaluation
MRI: Magnetic resonance imaging
NIA-AA: National Institute on Aging–Alzheimer's Association
pE: Pyroglutamate
p-tau: Phosphorylated tau
QC: Glutaminyl cyclase
SMC: Subjective memory complaints
VEGF: Vascular endothelial growth factor
