# Supplementary figures and images for: Glutaminyl cyclase activity correlates with levels of Aβ peptides and mediators of angiogenesis in cerebrospinal fluid of Alzheimer’s disease patients
Source: Alzheimers Res Ther. 2017 Jun 6;9:38. doi: 10.1186/s13195-017-0266-6 (PMC5461753; doi:10.1186/s13195-017-0266-6)

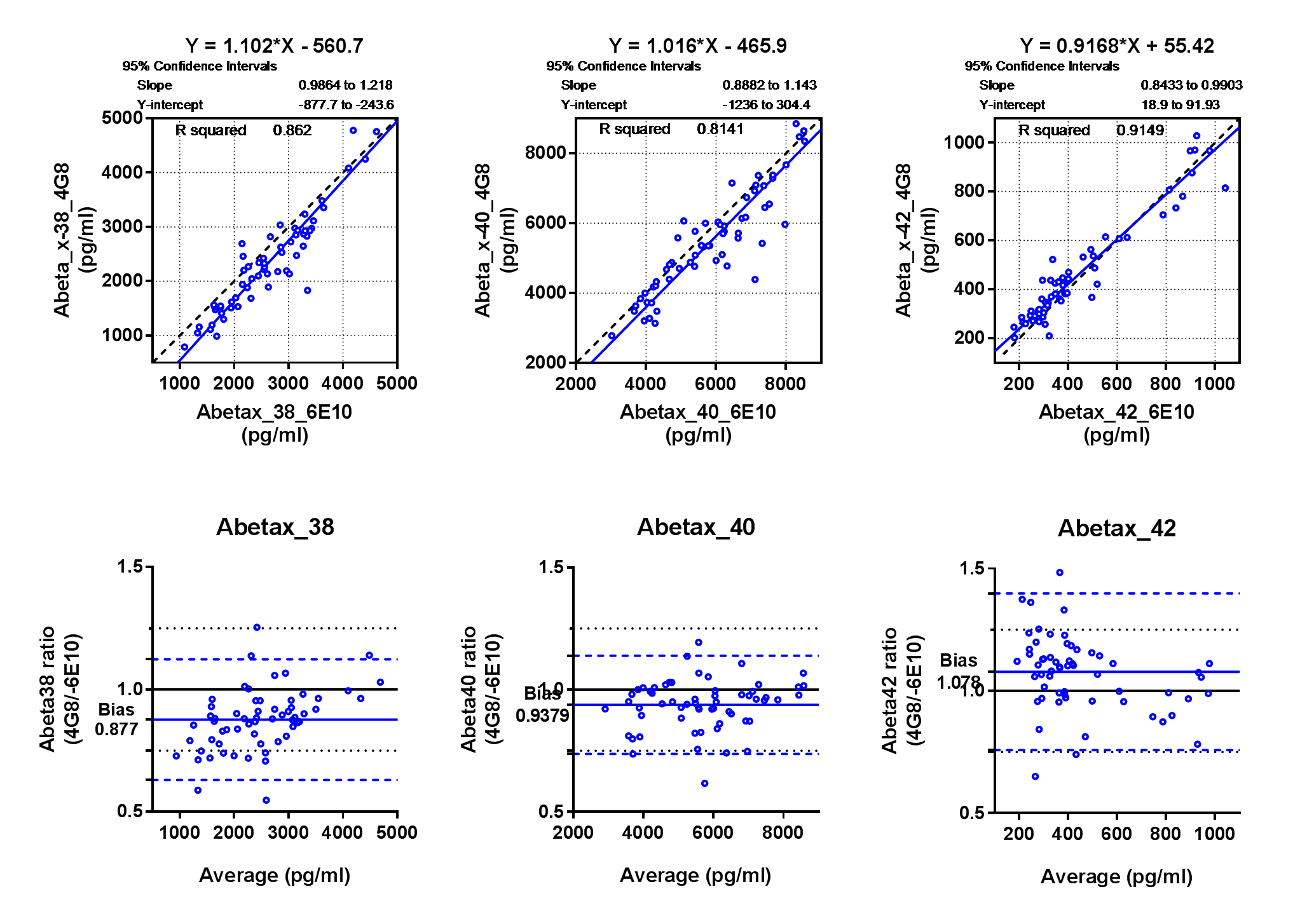

Supplement: Supplementary file 1 — showing method comparison for Aβs x-38, x-40 and x-42 determination in CSF of AD patients and SMC: MSD Aβ-6E10 versus MSD Aβ-4G8 multiplex. Upper panels: scatter plots with regression line (Deming regression, blue solid) and line of identity (black dashed). Regression equation with 95% confidence intervals of slope and intercept and coefficient of determination (R 2) given at top of each figure. Lower panels: Bland–Altman plots with line of identity (solid, black), bias (solid, blue), limits of acceptance (dashed, blue) and ±25% deviation between assays (dotted, black). Aβ38: regression line slope (1.10 (0.99, 1.22)) is slightly higher but not significantly different from 1, whereas intercept (−561 (−878, −244) pg/ml) is significantly below 0. Aβ 40: slope (1.02 (0.89, 1.14)) and intercept (−466 (−1236, 304) pg/ml) are not significantly different from 1 or 0, respectively. Regression line is parallel but slightly below the line of identity, resulting in slightly lower concentrations determined by the 4G8 assay (about 94% of 6E10 in mean). In the Bland–Altman plot, there is no trend over the concentration range analysed and only four samples show more than 25% lower values compared with the 6E10 assay. Aβ42: regression line slope of 0.92 (0.84, 0.99) is lower than 1 and the intercept of 55 (19, 92) pg/ml is significantly above 0. Therefore, in the low concentration range (<400 pg/ml), higher values of Aβ42 are determined with the 4G8 assay compared with the 6E10 assay. Because of the group difference, this affects most of the AD samples whereas for the control samples very similar values were determined with both assays (see Bland–Altman plot). (TIF 486 kb) [file 13195_2017_266_MOESM1_ESM.tif]

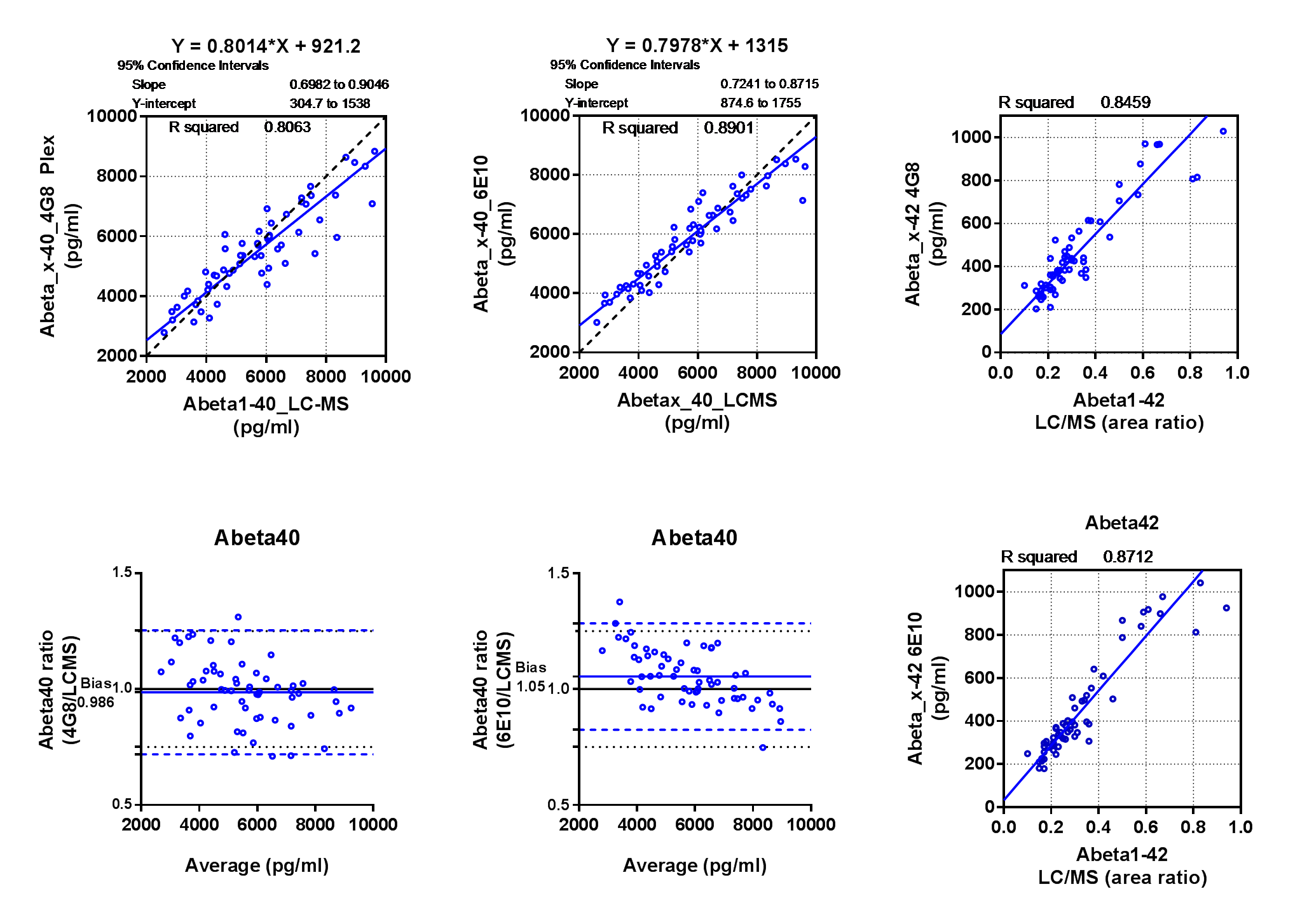

Supplement: Supplementary file 2 — showing method comparison for Aβ determination in CSF of AD patients and SMC: LC-MS (relative quantification) versus MSD Aβ multiplex. Upper panels: scatter plot with Deming regression lines. Lower panels, Bland–Altman plots for Aβ40 assay comparison. For Aβ1-42, only relative quantification was done with LC-MS. LC-MS determinations of Aβ(1–40) and Aβ(1–42) show correlation with both Aβ multiplexes. Coefficients of determination were found to be slightly better with the 6E10 multiplex assays. For the Aβ40 assays, Deming regression lines show slope < 1 and cross the lines of identity in the medium concentration range. Compared with the ELISAs, the LC-MS assay underestimates Aβ40 in the low concentration range and overestimates it at high concentrations. Nevertheless, mean deviation is <5% and there were only 5 out of 60 and 2 out of 60 measurements out of the ±25% range compared with the 4G8 multiplex or 6E10 multiplex, respectively. (TIF 484 kb) [file 13195_2017_266_MOESM2_ESM.tif]

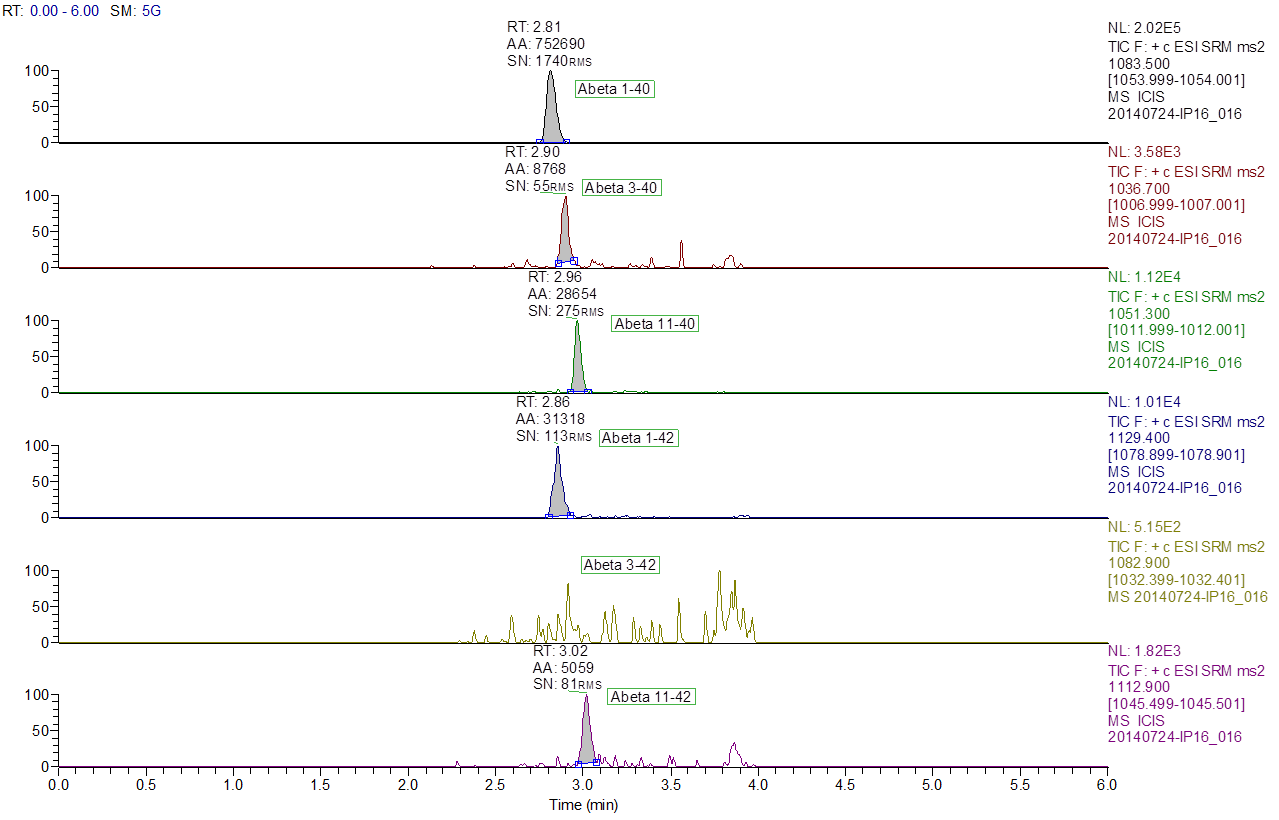


**Supplementary Figure 1**

Supplement: Supplementary file 4 — showing LC-MS/MS chromatogram of endogenous full-length peptides Aβ1-40 and Aβ1-42 and N-terminally truncated peptides Aβ3-40, Aβ11-40 and Aβ11-42 extracted from 100 μl CSF. Aβ3-42 was not detectable (see lane 5). (DOCX 85 kb) [file 13195_2017_266_MOESM4_ESM.docx]
