# Supplementary material for: Glutaminyl cyclase activity correlates with levels of Aβ peptides and mediators of angiogenesis in cerebrospinal fluid of Alzheimer’s disease patients
Source: Alzheimers Res Ther. 2017 Jun 6;9:38. doi: 10.1186/s13195-017-0266-6 (PMC5461753; doi:10.1186/s13195-017-0266-6)
Supplement: Supplementary file 3 — presenting a summary of the respective reference Aβ peptides (sequence, molecular weight, charge state, selected SRM transitions and provider. V* = Val (U13C5; 15 N)), and Table S2 presenting relative response of endogenous Aβx-40 peptides (analyte versus internal standard peak area ratios) extracted from a native CSF sample pool analysed over a storage period of 1 month at −80 °C (n = 3). (DOCX 40 kb) [file 13195_2017_266_MOESM3_ESM.docx]

Additional file 3

**Table S1**

**Table S2**
